# TCRep 3D: An Automated *In Silico* Approach to Study the Structural Properties of TCR Repertoires

**DOI:** 10.1371/journal.pone.0026301

**Published:** 2011-10-28

**Authors:** Antoine Leimgruber, Mathias Ferber, Melita Irving, Hamid Hussain-Kahn, Sébastien Wieckowski, Laurent Derré, Nathalie Rufer, Vincent Zoete, Olivier Michielin

**Affiliations:** 1 Multidisciplinary Oncology Center, Lausanne University Hospital (CHUV), Lausanne, Switzerland; 2 Swiss Institute of Bioinformatics (SIB), Lausanne, Switzerland; 3 Ludwig Institute for Cancer Research, Lausanne Branch, Epalinges, Lausanne, Switzerland; 4 The National Centre of Competence in Research (NCCR), Lausanne, Switzerland; 5 Department of Research, University Hospital Center and University of Lausanne, Lausanne, Switzerland; 6 Urology Research Unit, Department of Urology, Lausanne University Hospital (CHUV), Lausanne, Switzerland; University of Michigan, United States of America

## Abstract

TCRep 3D is an automated systematic approach for TCR-peptide-MHC class I structure prediction, based on homology and *ab initio* modeling. It has been considerably generalized from former studies to be applicable to large repertoires of TCR. First, the location of the complementary determining regions of the target sequences are automatically identified by a sequence alignment strategy against a database of TCR Vα and Vβ chains. A structure-based alignment ensures automated identification of CDR3 loops. The CDR are then modeled in the environment of the complex, in an *ab initio* approach based on a simulated annealing protocol. During this step, dihedral restraints are applied to drive the CDR1 and CDR2 loops towards their canonical conformations, described by Al-Lazikani *et. al.* We developed a new automated algorithm that determines additional restraints to iteratively converge towards TCR conformations making frequent hydrogen bonds with the pMHC. We demonstrated that our approach outperforms popular scoring methods (Anolea, Dope and Modeller) in predicting relevant CDR conformations. Finally, this modeling approach has been successfully applied to experimentally determined sequences of TCR that recognize the NY-ESO-1 cancer testis antigen. This analysis revealed a mechanism of selection of TCR through the presence of a single conserved amino acid in all CDR3β sequences. The important structural modifications predicted *in silico* and the associated dramatic loss of experimental binding affinity upon mutation of this amino acid show the good correspondence between the predicted structures and their biological activities. To our knowledge, this is the first systematic approach that was developed for large TCR repertoire structural modeling.

## Introduction

Recognition by the CD8+ T-cell receptor (TCR) of immunogenic peptide (p) presented by class I major histocompatibility complexes (MHC) is one key event in the specific immune response against virus-infected cells or tumor cells, leading to T-cell activation and killing of the target cell. Structural studies have revealed how the molecular recognition of pMHC by the TCR is mediated by six complementary determining regions (CDR) of the TCR at the interface with the pMHC complex. Each chain of the TCR (α and β) is bearing three loops called CDR1, CDR2 and CDR3. The CDR2 loops form the outside of the binding site, thus mainly contacting the alpha helices of the pMHC. CDR2 loops hence participate in the diagonal binding orientation that is generally observed on TCRpMHC structures [1]. CDR1 loops interact with the MHC but also contact the N- and C-termini of the peptide [2] [3] along with CDR3 that are the central loops in the TCR binding site and mostly interact with the peptide. However, the commonly accepted paradigm of CDR1 and CDR2 binding to the MHC and CDR3 to the peptide does not fully account for the true structural complexity of TCRpMHC complexes. Indeed, all CDR loops interact both with the peptide and MHC and their modeling should not favor peptide or MHC interactions regardless of the CDR studied [4].

CDR3 sequences are encoded by combination of gene elements, P- and N-region nucleotide addition and joining flexibility conferring a much greater diversity of lengths and sequences. The study of Al-Lazikani *et al.* [5] on existing TCRpMHC experimental structures revealed the existence of a limited number of canonical backbone conformations for CDR1 and 2 of both Vα and Vβ of the TCR. These canonical groups of CDR1 and CDR2 structures are identified by a combination of CDR length requirements and the presence of key residues at defined positions within the TCR sequences.

Experimental techniques used to determine the sequences of TCR that bind to a pMHC complex [6] have recently been used intensively, leading to the collection of large repertoires of TCR sequences that are specific for a given pMHC [7] [2]. In recent studies on the immunodominant human tumor antigen Melan-A(MART-1) [2] and on the NY-ESO-1 cancer testis antigen [7], restricted sets of T-cells were found to recognize the peptide/HLA-A*0201 pMHC complex. The TCR repertoire specific for the Melan-A decamer (ELAGIGILTV) was biased towards a Vα2.1 usage and that of NY-ESO-1 (SLLMWITQC) towards Vβ13, Vβ1 and Vβ8. To understand the selection mechanisms that underlie these restricted gene usage, there is a need for *in silico* approaches that take thorough advantage of the knowledge accumulated in TCRpMHC biology [8]. Such dedicated system may provide model structures that convey functional information and allow the identification of conserved 3D binding motifs that are not obvious from repertoire sequences alone.

Following the study of Michielin *et al.* [9], we set up an expert modeling method, called TCRep 3D, dedicated to the modeling of high quality TCRpMHC complexes, and focusing on the CDR loops structure. This approach has been designed to include optimal automation to analyze numerous TCR sequences and provide functional insight on the interaction between TCR and pMHC. It makes use of homology models of TCRpMHC [10] [9], based on the constantly increasing list of available crystal structures that have been solved since the first one in 1996 [11] and are available in the Protein Data Bank [12] (http://www.rcsb.org/). Importantly, we developed in this study a dedicated method for systematic *ab initio* refinement of the six CDR loops using a simulated annealing approach. This method is based on the fact that hydrogen bonds between the TCR and the pMHC are known to be of major importance for the TCRpMHC complexation and protein-protein interaction [13]. Such potential bonds were intensively searched during this step of the modeling, by iteratively generating conformers of a CDR loop, and by including new restraints derived from the hydrogen bonds statistics of the previous iterations in the subsequent ones. The canonical loop information [5] is also accounted for by means of additional restraints automatically derived by our program. Our approach does not favor explicitly the CDRs to contact either the peptide or the MHC, since all CDR – pMHC contacts are equally considered.

We used a test set of 10 known crystal structures to assess the efficiency of the *ab initio* CDR prediction method according to its ability to reproduce CDR loop conformations and crystal contacts. The accuracy of our approach was then compared to other selection methods based on several popular scoring functions (Anolea [14], Dope [15] and the Modeller scoring function [10]).

Ultimately, the modeling of 6 TCRpMHC structures from experimental sequences related to the NY-ESO-1 TCR repertoire revealed a striking mechanism of selection through the presence of a single conserved Gly situated in the center of all CDR3β. An *in vitro* experimental functional study of mutations of this amino acid combined with *in silico* modeling of several mutants was performed. It confirmed that dramatic predicted structural changes caused by these mutation are linked to the loss of affinity of the TCR to NY-ESO-1/HLA-A*0201.

## Results

**Figure 1:**
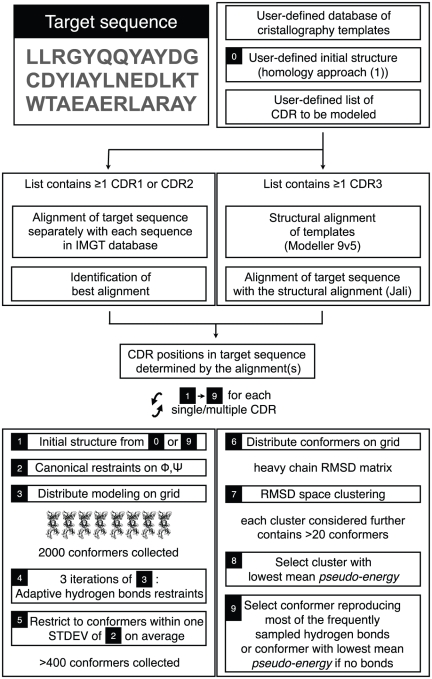
TCRpMHC modeling general procedure. Key steps are numbered in black boxes and referenced to in the Materials and Methods section.

Figure 1 shows the detailed modeling procedure. In the following, Root Mean Square Deviations (RMSD) are calculated over heavy atoms, unless specified otherwise.

### CDR loops prediction

We first assessed the capacity of the *ab initio* prediction (Figure 1) to model a single CDR loop in its crystallographic environment, bound to pMHC. This approach is referred to as the single-loop approach. Each CDR loop from 10 available TCRpMHC crystal structures was modeled (see Table 1) using the *ab initio* prediction and the crystal structure as the initial loop conformation. A total of 60 CDR loops of different lengths were computed (CDR1 length: 8 to 10 amino acids, CDR2: 5 to 7 and CDR3: 3 to 11). 82% of the predicted CDR had a RMSD from the crystal structure below the 3.0 Å threshold used to define successful predictions (see Discussion). The average RMSD was 2.21 Å (Table 1). Hence, single CDR were successfully predicted in the environment provided by the crystal structure. During this test, we verified that the sampling (see Methods) was not confined in the starting local minimum and artificially biased towards the reference structure, i.e. that no memory effect exists. For this, we computed the RMSD between the starting structure and the first CDR conformer for each CDR. An average of 3.70 Å (SD = 1.68) was obtained, which confirmed that the exploration of the conformational space was effective from the beginning of the simulation.

**Table 1:** RMSD, in Å, calculated for each CDR of the test set of 10 crystal structures, for independent and sequential *ab initio* loop modeling.

		Model vs crystal root mean square deviation [Å]						
PDB ID		CDR2β	CDR1β	CDR2α	CDR1α	CDR3α	CDR3β	Average (SD)
**1ao7**	Number of residues	*7*	*8*	*5*	*9*	*8*	*9*	
	Independent loops modeling	0.66	1.62	1.88	2.27	1.72	2.42	1.76 (0.62)
	Sequential loops modeling	0.66	1.60	1.88	3.37	1.94	2.74	2.03 (0.94)
**1bd2**		*7*	*8*	*6*	*9*	*6*	*8*	
	"	0.56	2.93	1.43	2.54	2.77	2.89	2.19 (0.97)
		2.29	1.64	1.61	2.78	1.26	3.65	2.21 (0.89)
**1g6r**		*7*	*8*	*6*	*9*	*6*	*8*	
	"	1.44	1.51	1.41	1.96	0.93	1.36	1.44 (0.33)
		1.44	1.62	1.40	2.57	0.89	3.80	1.95 (1.06)
**1kj2**		*7*	*9*	*6*	*9*	*7*	*11*	
	"	1.19	3.98	1.11	1.61	1.14	4.11	2.19 (1.45)
		1.28	4.07	1.68	2.32	3.50	6.04	3.15 (1.77)
**1lp9**		*7*	*8*	*5*	*9*	*9*	*5*	
	"	1.26	1.42	1.96	2.28	1.26	2.62	1.80 (0.58)
		1.26	1.73	2.19	2.80	1.48	4.39	2.31 (1.16)
**1mi5**		*7*	*8*	*6*	*10*	*10*	*6*	
	"	4.79	2.29	1.55	2.61	4.45	1.58	2.88 (1.41)
		4.81	1.46	3.49	2.81	5.64	2.02	3.37 (1.61)
**1nam**		*7*	*9*	*7*	*10*	*10*	*7*	
	"	3.00	4.54	2.62	3.22	2.80	6.21	3.73 (1.39)
		3.09	4.66	1.54	4.41	2.74	6.38	3.80 (1.7)
**1oga**		*7*	*8*	*6*	*8*	*7*	*5*	
	"	1.15	1.86	2.66	0.93	3.83	1.14	1.93 (1.13)
		1.24	1.86	2.61	0.95	3.80	1.24	1.95 (1.08)
**2ckb**		*7*	*8*	*6*	*9*	*6*	*3*	
	"	1.56	1.78	2.54	3.09	1.41	1.37	1.96 (0.7)
		1.29	1.23	2.21	2.20	1.13	1.76	1.64 (0.49)
**2bnr**		*7*	*8*	*6*	*9*	*9*	*7*	
	"	1.12	1.86	2.80	2.24	2.16	3.35	2.26 (0.77)
		1.24	1.40	2.53	3.09	3.64	2.65	2.43 (0.94)
**Average**		*7.0 (0)*	*8.2 (0.4)*	*5.9 (0.54)*	*9.1 (0.54)*	*7.8 (1.54)*	*6.9 (2.17)*	
**(SD)**	"	1.67 (1.22)	2.38 (1.03)	2.00 (0.59)	2.28 (0.64)	2.25 (1.13)	2.71 (1.49)	2.21 (1.12)
		1.86 (1.18)	2.13 (1.14)	2.11 (0.61)	2.73 (0.84)	2.60 (1.45)	3.47 (1.65)	2.48 (1.32)

**Figure 2:**
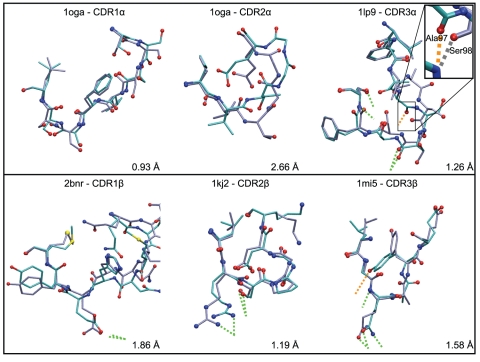
A selection of CDR structures successfully modeled by the single-loop approach in the *ab initio* prediction. Experimental structures (purple) are superimposed to CDR models (cyan). Oxygen, nitrogen and sulfur atoms are colored in red, blue and yellow, respectively. Dotted lines show hydrogen bonds between CDR and pMHC. Hydrogen bonds reproduced by the model in green and in orange otherwise. In the case of 1lp9 CDR3, the hydrogen bond with pMHC which is not reproduced (involving Ala97), is replaced in the model by another hydrogen bond involving the carbonyl group of the Ser98 backbone (additional contact in Table 2).

Two CDR3 loops showed a RMSD to crystal above 5 Å: 1fo0 CDR3α with 5.95 Å and 1nam CDR3β with 6.21 Å. Interestingly, the structural analysis of the 1fo0 crystal demonstrated that a hydrogen bond is present between the hydroxyl group of the Tyr97 residue of CDR3α and the backbone carbonyl of Ala135 of a neighbor MHC molecule in the crystal. This crystal contact apparently deviates the CDR3 away from the pMHC in the experimental structure. When 1fo0 CDR3α loop is modeled without the crystal environment, it adopts a conformation directed towards the peptide as a direct consequence of the use of iterative hydrogen bonds restraints during the simulated annealing procedure (see Methods and Figure S1). 1fo0 was hence not considered further in this study. Figure 2 shows successful predictions for six illustrative loops computed in the single-loop approach, both in terms of RMSD from the experimental structure and hydrogen bonds reproduction.

We tested the ability of the *ab initio* prediction to model all 6 CDR of each TCR crystal in a successive-loops approach, a scenario corresponding to the real application (Figure 1). The CDR were modeled in the following order: CDR2β, CDR1β, CDR2α, CDR1α and finally both CDR3 together. The choice of this sequence was devised to model first the CDR in the periphery of the TCR binding site, since they generally do not play the key role in TCR-peptide recognition, as opposed to CDR3 loops [16]. Once the CDR2β has been predicted, its conformation is kept fixed during the subsequent optimization of CDR1β and so on with the other CDR in the order mentioned above. This successive-loops approach showed a success rate of 72% compared to 82% for the single-loops scenario (see Table 1). The average RMSD from the crystal structures was 2.48 Å (SD = 1.32) compared to 2.21 Å (SD = 1.12) for the single-loop approach. Interestingly, we reported that an incorrectly predicted CDR loop did not systematically lead to a failure for the modeling of subsequent loops. Indeed, the RMSD for 1mi5 CDR1α and β were 2.81 Å and 1.46 Å, respectively, while the RMSD for the CDR2α and β modeled in the previous step were 3.49 Å and 4.81 Å, respectively (Table 1). This illustrates the robustness of the algorithm with respect to the accuracy of the loop environment. Numerical data for all loops computed both by single-loop and successive-loop approaches are given in Table 1 and Table 2.

**Table 2:** Hydrogen bonds statistics in the test set for independent and sequential *ab initio* loop modeling.

		Hydrogen bonds between TCR and pMHC : reproduced/total (additionnal)						
PDB ID		CDR2β	CDR1β	CDR2α	CDR1α	CDR3α	CDR3β	Total
**1ao7**	Number of residues	*7*	*8*	*5*	*9*	*8*	*9*	
	Independent loops modeling	0/0 (0)	1/1 (0)	0/0 (1)	1/2 (2)	5/5 (1)	2/3 (1)	9/11 (5)
	Sequential loops modeling	0/0 (0)	1/1 (0)	0/0 (1)	0/2 (4)	1/5 (1)	0/3 (0)	2/11 (6)
**1bd2**		*7*	*8*	*6*	*9*	*6*	*8*	
	"	0/0 (0)	0/0 (3)	1/1 (0)	1/1 (1)	2/3 (1)	0/1 (1)	4/6 (6)
		0/0 (0)	0/0 (1)	1/1 (0)	1/1 (0)	2/3 (2)	0/1 (2)	4/6 (5)
**1g6r**		*7*	*8*	*6*	*9*	*6*	*8*	
	"	0/0 (1)	1/1 (3)	0/0 (2)	0/2 (1)	2/2 (1)	0/0 (1)	3/5 (9)
		0/0 (1)	0/1 (3)	0/0 (2)	0/2 (2)	2/2 (0)	0/0 (1)	2/5 (9)
**1kj2**		*7*	*9*	*6*	*9*	*7*	*11*	
	"	2/2 (1)	0/0 (4)	0/0 (0)	1/1 (1)	3/3 (1)	1/1 (6)	7/7 (13)
		2/2 (1)	0/0 (4)	0/0 (2)	1/1 (1)	2/3 (2)	0/1 (5)	5/7 (15)
**1lp9**		*7*	*8*	*5*	*9*	*9*	*5*	
	"	0/0 (0)	1/1 (0)	0/0 (1)	0/0 (1)	3/4 (4)	0/0 (1)	4/5 (7)
		0/0 (0)	1/1 (0)	0/0 (2)	0/0 (1)	2/4 (4)	0/0 (0)	3/5 (7)
**1mi5**		*7*	*8*	*6*	*10*	*10*	*6*	
	"	2/2 (3)	0/0 (0)	0/0 (1)	2/2 (0)	0/1 (5)	3/4 (2)	7/9 (11)
		2/2 (2)	0/0 (0)	0/0 (2)	1/2 (1)	0/1 (6)	1/4 (3)	4/9 (14)
**1nam**		*7*	*9*	*7*	*10*	*10*	*7*	
	"	0/0 (2)	0/0 (2)	0/0 (1)	0/1 (4)	1/2 (1)	0/0 (3)	1/3 (13)
		0/0 (1)	0/0 (2)	0/0 (1)	0/1 (2)	1/2 (2)	0/0 (1)	1/3 (9)
**1oga**		*7*	*8*	*6*	*8*	*7*	*5*	
	"	2/2 (0)	1/1 (0)	0/0 (0)	0/0 (0)	0/0 (5)	4/4 (2)	7/7 (7)
		2/2 (0)	1/1 (0)	0/0 (0)	0/0 (0)	0/0 (5)	2/4 (1)	5/7 (6)
**2ckb**		*7*	*8*	*6*	*9*	*6*	*3*	
	"	0/0 (2)	0/0 (4)	0/0 (2)	1/2 (3)	1/2 (2)	0/0 (1)	2/4 (14)
		0/0 (3)	0/0 (3)	0/0 (2)	2/2 (3)	1/2 (2)	0/0 (0)	3/4 (13)
**2bnr**		*7*	*8*	*6*	*9*	*9*	*7*	
	"	1/1 (0)	1/1 (0)	1/1 (1)	0/0 (0)	2/4 (2)	2/2 (4)	7/9 (7)
		1/1 (0)	1/1 (0)	1/1 (1)	0/0 (2)	0/4 (1)	2/2 (3)	5/9 (7)
**Total**		*-*	*-*	*-*	*-*	*-*	*-*	
	"	7/7 (9)	5/5 (16)	2/2 (8)	6/11 (13)	19/26 (23)	12/15 (22)	51/66 (92)
		7/7 (8)	4/5 (13)	2/2 (13)	5/11 (16)	11/26 (25)	5/15 (16)	34/66 (91)

**Figure 3:**
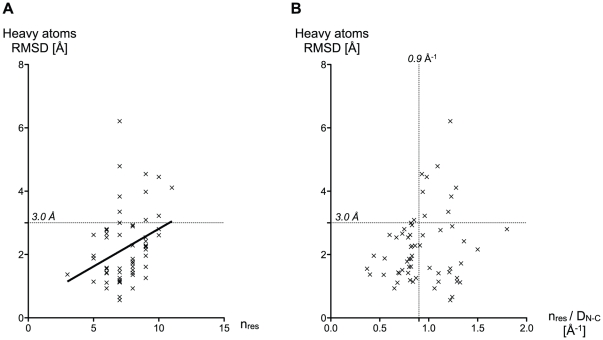
RMSD of all predicted single-loops of the test set plotted against two parameters. (A) RMSD against the number of residues n that form the loop, (B) RMSD against the n/D_N-C_ value of the loop in Å^−1^. The ratio n/D_N-C_, is a good *a priori* indicator of modeling success.

At the sequence level, very few CDR properties could help predict the success or the failure of our structure prediction algorithm. Nevertheless, CDR length is a useful indicator (Figure 3A). As could be observed, RMSD values between predictions and their respective crystal references slightly increased in average, with the loop length. A *n/D_N-C_* score was defined as the ratio between the number of residues that form the loop, n, and the distance between the N-terminal and C-terminal ends of the CDR, D_N-C_. This score describes the «elongation» of the backbone of the CDR: small values of *n/D_N-C_* correspond to elongated loops, and large values to curved ones. It reflects the size of the accessible conformational space for a loop of a given number of residues, which is expected to be larger for curved loops. Considering our 3.0 Å success criteria for RMSD (see Discussion), Figure 3B shows that a CDR loop is likely to be correctly predicted *ab initio* when its *n/D_N-C_* is lower than 0.9 Å^−1^. The 0.9 cutoff still retained 50% of the cases present in the test set, whereas the cutoff based on the number of residues alone (loops that are no longer than 6 residues are correctly predicted) retained less than 30% (Figure 3A). Despite its limitations, the *n/D_N-_*_C_ is thus a better descriptor than n alone, to identify the cases likely to be correctly predicted. For larger values of *n/D_N-C_*, the quality and the reliability of the prediction cannot be assessed *a priori*.

### Potential hydrogen bonds identification

The biological function of a TCR depends on its affinity for the peptide-MHC complex [17], [18] [19]. This affinity is, in turn, a function of the interactions taking place at the TCRpMHC interface, and in particular of the hydrogen bonds [13]. Therefore, the modeling approach was specifically designed to progressively restrain the exploration of the conformational space to regions of high occurrence of hydrogen bonds between the TCR and the pMHC (see Methods).

An analysis of the structures of CDR predicted by the single-loop and successive-loops modeling approaches showed that the final models reproduced 77% and 52% of the total 66 hydrogen bonds present in the crystal structures, respectively (Table 2). The performance of TCRep 3D in hydrogen bonds reproduction is in reasonable qualitative agreement with the RMSD from the experimental structure. Indeed, among the loops that were predicted with a RMSD lower than 3.0 Å from the experimental structure, 83% and 59% of the potential hydrogen bonds were reproduced by the single and successive CDR modeling, respectively (see Table 1 and Table 2).

Interestingly, the approach performed differently on loops with no hydrogen bond in the crystal. Indeed, all the CDR with no hydrogen bond with the pMHC in the reference crystal showed on average 1.33 (SD = 1.33) potential hydrogen bonds identified in the successive-loops approach. This number was significantly higher for the CDR loops showing hydrogen bounds in the crystal structure: 2.70 (SD = 1.57, p<0.001). An average of 13.2 potential hydrogen bonds (SD = 10.8) were identified during the sampling of a given CDR loop in the last iteration (see methods). It is noteworthy however that 78% of the hydrogen bonds present in the crystal were actually observed among the 6 most frequent ones sampled on each CDR.

### Iterative sampling and scoring quality

The important novel aspects of TCRep 3D are the systematic use of canonical restraints and hydrogen bonds derived restraints during iterative loop samplings and the use of a scoring function based on the sampled hydrogen bonds (see Materials and Methods). The efficiency of the *ab initio* prediction to produce an optimal model was compared to standard approaches and our scoring function was compared to several well established energy scoring methods: Anolea [14], Dope [15] and the Modeller *pseudo*-energy [10] scoring functions.

**Figure 4:**
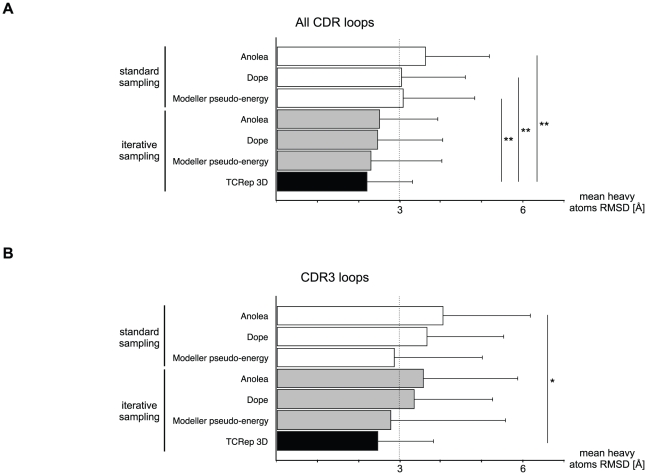
TCRep 3D performs better than common modeling approaches because of iterative hydrogen bonds sampling. Single-loop test set modeling with Anolea, Dope or Modeller pseudo-energy scoring functions with and without iterative hydrogen bond sampling compared to TCRep 3D. (A) All-loops average RMSD of models to crystal structures. (B) CDR3 loops average RMSD. (*: p-value<0.05, **: p-value<0.01).

Starting from the crystal structures, the CDR were independently modeled without adding restraints and a standard set of 2000 conformers with a Modeller *pseudo*-energy function value lower than 500 was collected for each CDR loop. The energy of each conformer was then computed, using the Anolea, Dope and the Modeller scoring functions. For each scoring function, we selected the conformer with the lowest energy as a final model. The average RMSD of the 60 single loops selected among a set of 2000 structures generated were computed for each function (Figure 4A). The average RMSD values were respectively 3.64 Å (SD = 1.57), 3.05 Å (SD = 1.57) and 3.09 Å (SD = 1.76). The use of these scoring functions after the iterative H-bonds sampling as implemented in TCRep 3D improved the average RMSD (2.52 Å (SD = 1.43), 2.47 Å (SD = 1.60) and 2.31 Å (SD = 1.75), respectively), in comparison to TCRep 3D which produced the best average RMSD value at 2.21 Å (SD = 1.12). Interestingly, our iterative sampling algorithm brought the average RMSD below the 3.0 Å cutoff irrespective of the scoring function. TCRep 3D performed significantly better than unrestrained simulated annealing with Anolea, Dope or Modeller scoring functions (p<0.001, p<0.005, p<0.0001, respectively). We identified for each loop, the element in the set of 2000 conformers with the lowest RMSD from the crystal; the corresponding RMSD average value over the 60 CDR was 1.24 Å (SD = 0.43) for the standard set and 1.23 Å (SD = 0.66) for the iterative set (i.e. modeled with restraints, see Methods).

Since the longest CDR loops, and also the most important loop modeling failures were contained in the CDR3 set (see Table 1), the same analysis restricted to CDR3 only was performed. It showed slightly higher average RMSD with Anolea, Dope or Modeller (4.07 Å (SD = 2.14), 3.68 Å (SD = 1.88) and 2.88 Å (SD = 2.17), respectively) (Figure 4B). Results improved after hydrogen bonds iterative sampling, with average RMSD of 3.36 Å (SD = 1.92), 3.59 Å (SD = 2.31) and 2.79 Å (SD = 2.81) respectively. Again, with an average RMSD of 2.48 Å (SD = 1.38), our algorithm remained below the 3.0 Å threshold with better performance (p<0.399, p<0.066, p<0.021 respectively). The average RMSD in the standard and iterative sets were 1.26 Å (SD = 0.44) and 1.34 Å (SD = 0.66), respectively, for the lowest RMSD selection restricted to CDR3. In summary, these results showed that TCRep 3D outperforms significantly standard methods in producing relevant loops conformations.

### A key Gly on CDR3β of NY-ESO-1 specific TCR

**Figure 5:**
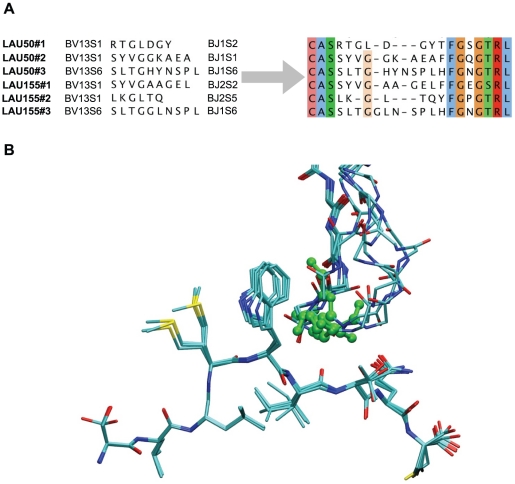
Sequence and structural models of the experimental set of CDR3β specific to NY-ESO-1/HLA-A2. (A) TCRVβ sequences of the experimental set of TCR, and structural alignment of the CDR3β, based on our structure predictions allow the identification of a conserved central Gly residue (conserved residues are colored). (B) Structural superposition of the peptide and the CDR3β of the six predicted TCR structures bound to NY-ESO-1/HLA-A2 visually confirm the key position of the central Gly of each CDR3β (green) for the CDR *lock* conformation around the peptide's Met4 and Trp5.

NY-ESO-1_157–165_ is one of the most important tumor antigen in melanoma [20] and is currently being used in many clinical trials. Analysis of the TCR repertoire selected in these patients has provided us with a large number of sequence data for which structural interpretation is needed [7]. These sequences were identified from naturally occurring HLA-A*0201/NY-ESO-1_157–165_–specific CD8+ T cells from five melanoma patients. Among them, LAU 155#1 TCR has a sequence identical to that of the experimental structure Vα23-Vβ13 TCR bound to NY-ESO-1/HLA-A2 (PDB code 2bnr [21]), except for 5 residues situated on the CDR3α (Gln95, Thr96, Ala97 instead of Thr95, Ser96, Asn97) and CDR3β (Ala97, Ala98 instead of Asn97, Thr98). In total, 6 TCR sequences showed a TCRVβ13 gene usage (CDR3 sequences and encoding gene numbers are shown in Figure 5A). Considering this, we focused on them and used the procedure described by Michielin *et. al.* [9] to build homology models of our target sequences using the 2bnr TCRpMHC complex along with TCR structures of our data set (see Methods and Table S1) as templates. This constrained the TCR binding orientation over the pMHC according to the known specific diagonal binding mode of Vα23-Vβ13 TCR bound to NY-ESO-1 [22]. As a consequence, very small structural deviations were observed between the pMHC in the models and the structure of 2bnr. The largest calculated RMSD between one of our models and the pMHC crystal structure was 1.19 Å. In particular, although the structure of the peptide showed small variations, especially on side chains (see Figure 5B), its overall position in the model was very close to that in the crystal structure, with a RMSD lower than 1.0 Å after superposition of the pMHC. We then applied our TCRep 3D successive-loops modeling algorithm to the homology-derived models to obtain a detailed analysis of TCR-pMHC interactions within this subset of the NY-ESO-1 TCR repertoire.

All final models reproduced the binding motif of CDR3 α and β making a *lock* around the two central residues Met4 and Trp5 of the peptide as described by Sami et al. [22] [7]. A structural alignment of the six models was performed and revealed a conserved central position for a Gly residue in the CDR3β sequence (Figure 5A, the central Gly is slightly shifted for LAU 50#1). Indeed, this Gly is remarkably conserved in all six CDR3β sequences and was shown to fit structurally into a notch under the peptide Trp5 side chain (Figure 5B).

**Figure 6:**
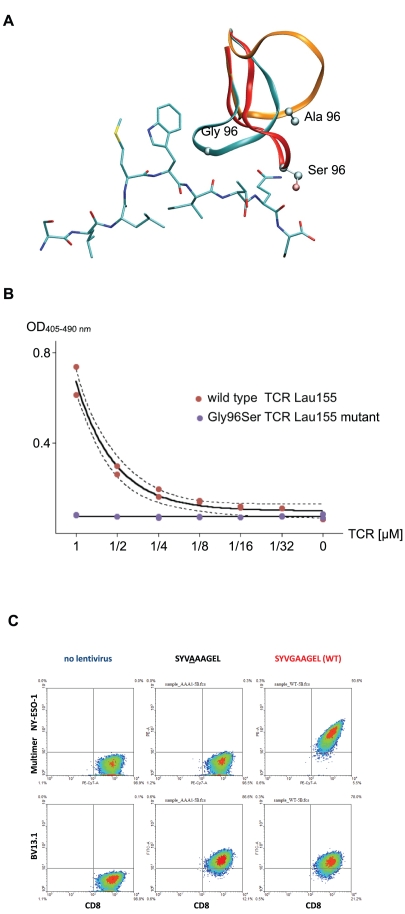
*In silico* and experimental mutation results in NY-ESO-1 repertoire. Mutations confirm the importance of the central Gly in CDR3β. (A) Dramatic structural rearrangements of predicted structures of mutated Gly96Ala (orange) and Gly96Ser (red) loops superposed with the non-mutated predicted structure of the CDR3β of LAU 155#1 (cyan). (B) Duplicated experimental titration ELISA on LAU 155#1 TCR and Gly96Ser mutant shows the loss of affinity resulting from these mutations. (C) Mutation of the Gly96 to Ala in CDR3β induced loss of binding of NY-ESO-1 multimer, as seen at the surface of SupT1 cells transduced with lentiviral particles.

We further investigated this pattern via *in silico* mutations from Gly to Ala and Ser. We repeated the coupled CDR3 α and β *ab initio* modeling on the six models. Modeling results showed a dramatic conformational change of the CDR3β. For example, the RMSD from the wild type CDR3β, calculated for the backbone atoms, were 5.88 Å (Gly to Ala) and 3.28 Å (Gly to Ser) for LAU 155#1 CDR3β (Figure 6A). This was confirmed on all the six mutated structures, for both Ala and Ser mutations with backbone RMSD values ranging from 3.09 Å to 5.88 Å. All mutated loops were unable to fit into the peptide notch.

Experimental mutations of the LAU 155#1 TCR Gly96 confirmed the importance of this central Gly in the CDR3β sequence *in vitro*. Soluble wild type and Gly96 to Ser mutant TCR, and NY-ESO-1/HLA-A*0201 pMHC were produced. Figure 6B shows the dramatic loss of binding affinity for the Gly96 to Ser mutant measured by titration ELISA experiment. Additionally, Gly96 to Ala mutated TCR was expressed at the surface of the T-cell line SUP-T1 using lentiviral vectors, and binding of NY-ESO-1/pMHC multimer, as measured by flow cytometry, was also completely lost (see Figure 6C).

## Discussion

There is a considerable interest amongst immunology groups studying T-cell biology to obtain functional information from TCR repertoire sequences. We set up an automated and dedicated system to model TCRpMHC complexes, focused on modeling the interface of the complex, especially hypervariable CDR loops. Clearly, only simulations of TCR bound to pMHC were performed in order to identify binding motifs governing the definition of TCR repertoires. The method presented in this paper showed a high efficiency and robustness for the prediction of TCRpMHC interfaces. Our method relies on using canonical group knowledge for CDR1 and CDR2 loops, successively refining CDR loops, iteratively looking for hydrogen bonds at TCR - pMHC interface and clustering simulated annealing models to select the best TCRpMHC model. Identifying CDR interactions with pMHC is key to understand the mechanisms of TCR selection while the models generated may provide optimal initial conditions for further TCR engineering [23].

### Canonical restraints and sampling quality

We expanded the canonical restraints on backbone dihedral angles described by Al-lazikani *et al.* [5] with new crystallographic data (Table S2). Canonical restraints were defined by their average values and standard deviations (SD). The restraint violation computed by Modeller depends crucially on the SD value [10]; the smaller the SD, the larger the violation. The efficiency of such dihedral angles restrictions was confirmed in *ab initio* simulations. As an example, Figure S2 shows the Ramachandran plots of the Arg residue at position 2 of CDR2β of 1kj2, for both unrestrained and restrained simulations. Restraints of Al-lazikani group β2-2 were used, i.e. ϕ = −106.71° with SD = 24.97° and ψ = 44.97° with SD = 97.68°. As expected, the ϕ and ψ angles sampled in the restrained simulation correspond to those defined by the canonical restraints (i.e. −160°≤ϕ≤−70° and −180°≤ψ≤180°). It is noteworthy that the large SD of ψ values in the canonical restraint implies that all ψ values can be sampled, as it was actually observed, while the ϕ angle is effectively restricted, according to the corresponding SD. In the region of the Ramachandran plot defined by the restraints, the sampling performed in the restrained simulation is comparable to that of the unrestrained one; the entire allowed region is well sampled. This confirms that the loops were not confined in a few narrow local energy minima during the production of conformers with canonical restraints. The application of restraints on the accessible conformational space of the CDR also successfully prevented the system to reach energetically unfavorable conformations and restricted simulations to more relevant regions of space (data not shown). Technically, this resulted in a significant gain in computational time. Indeed, the time required to collect 2000 CDR conformers with a Modeller *pseudo*-energy function below a cutoff of 500 (see methods) was divided by (up to) 3 when canonical restraints were available.

### RMSD cutoff

**Figure 7:**
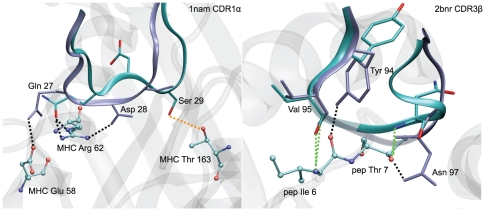
Adequacy of 3.0 Å RMSD cutoff. Structural and hydrogen bond inspection confirm the modeling failure of 1nam CDR1α, RMSD 3.22 Å, while 2bnr CDR3β, RMSD 3.35 Å, may be considered a successful prediction. Structural superposition of predicted loops (cyan ribbon) for 1nam CDR1α (A) and 2bnr CDR3β (B) with their respective crystal references (purple ribbon). Dotted lines: CDR-pMHC potential hydrogen bonds made between the CDR and the pMHC, green: potential hydrogen bonds reproduced by the predicted loop, orange: potential hydrogen bonds observed in the crystal structure but not in the prediction and black: additional potential hydrogen bonds of the modeled CDR. pMHC residues making hydrogen bonds with the CDR are explicitly shown in ball and stick. Peptide and MHC are shown in ribbon representation, in transparent grey.

For the evaluation of loop modeling successes, we used an empiric heavy atom RMSD cutoff of 3.0 Å. The structure similarity between predictions and experimental conformations, i.e. reproduction of the global conformation and native contacts, was satisfying according to systematic visual inspections of all superimposed structures (see results and Figure 2). To test the influence of the RMSD cutoff value on the success rate (see results), a 3.5 Å cutoff was considered, for which the success rates were 88% for single-loops and 82% for successive-loops procedures compared to 82% and 72%, respectively, for the 3.0 A cutoff. Nevertheless, for cases corresponding to RMSD between 3 Å and 3.5 Å, the quality of the prediction was not satisfying for all CDR: in some cases, the method was not able to reproduce the experimental hydrogen bonds, but created alternative ones, while the conformation was strongly altered compared to the crystal structure, see below, Table 1, Table 2 and Figure 7. Although 1nam CDR1α and 2bnr CDR3β have heavy atoms RMSD values of 3.22 Å and 3.35 Å, respectively, the prediction could be considered a success in the second case, but not in the first one, showing that a 3.5 Å cutoff does not separate properly successes and failures. As a consequence, the more stringent RMSD cutoff of 3.0 Å was used in this study.

### Intensive hydrogen bonds search

Because the creation of hydrogen bonds between CDR loops and MHC is an important mechanism that governs the selection of a TCR repertoire [13], our approach incorporated a novel iterative strategy to converge towards such favorable interaction pattern between TCR and pMHC. The efficiency of the hydrogen bonds sampling generally increased through the application of hydrogen bonds derived restraints, compared to a modeling without iterations (data not shown).

**Figure 8:**
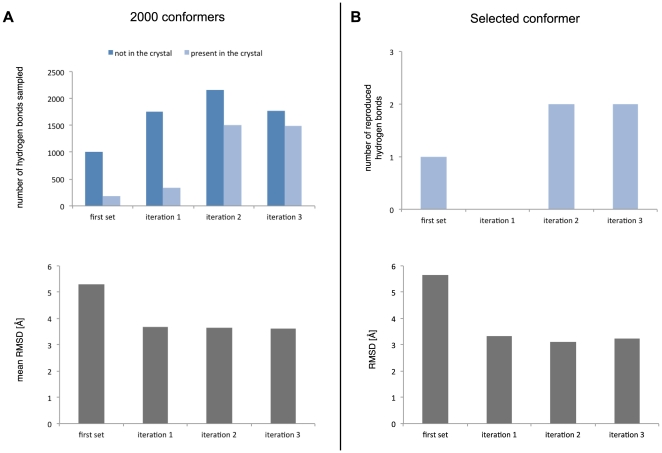
Iterative application of hydrogen bonds derived restrains improves simulation results. The example of 1kj2 CDR3α in sequential loop modeling demonstrates for the complete set of 2000 conformers (A) increased number of hydrogen bonds sampled with larger proportion of interactions present in the reference crystal and decreased mean RMSD at each iteration. (B) Statistics of the best conformers at each iteration. Three iterations were sufficient to obtain optimal results.

As an illustration, the impact of this strategy on the modeling of 1kj2 CDR3α, a difficult loop to model (*n/D_N-C_* = 1.1 Å^−1^), is presented in Figure 8. On average, the RMSD of the conformers decreased from 5.31 Å (SD = 1.68) initially to 3.61 Å (SD = 1.34) after three iteration steps. In the complete set of 2000 conformers, the total number of sampled hydrogen bonds and the proportion of hydrogen bonds present in the reference crystal increased. After three iterations, the final conformer had 2 out of the 3 hydrogen bonds present in the reference structure, while its RMSD initially at 5.66 Å reached 3.23 Å. Increasing the number of iterations to 4 in that example did not improve further the collection of hydrogen bonds. For the general approach, 3 iterations were made, since this provided the best compromise between modeling improvement and required CPU time (data not shown).

This approach led to the identification of numerous potential hydrogen bond contacts, with many not observed in the crystal structures. Obviously, the crystal structure of a TCRpMHC provides an average conformation of the molecules in the crystal state and does not describes comprehensively its dynamical behavior at room temperature in solution.

### Successive-loops modeling

The single-loops approach was set up to assess our ability to model a loop in the correct environment provided by the known crystal structure. The method gave a satisfying success rate of 82% using the 3.0 Å cutoff discussed earlier, illustrating the efficiency of the loop modeling procedure when the environment of the modeled part is correct. The success rate decreased to 72% for the successive-loops approach. The performance change can be explained by the accumulation of errors during the successive modeling of the loops; indeed errors during CDR2 modeling will impact the CDR1 modeling, whose error will, in turn, impact the CDR3 modeling. Successive-loops modeling of the CDR is however required in the general case since a standard homology modeling procedure cannot provide reliable structures for all CDR as TCR templates are in limited number and CDR conformations may differ from unbound to bound states [9] [22] [24] [25]. It is worth noting that, in our approach, the conformational space that is explored by CDR loops is much larger than the amplitude of CDR conformational change upon binding. And since there is no memory effect during the *ab initio* modeling (see Results), our results are not influenced by the amplitude of the conformational change of the CDRs upon binding (Figure S3).

The CDR refinement starts from the periphery of the binding site, to end with central CDR3. In this manner, the most reliable environment can be predicted for the central CDR3 that interact the most with the rest of the CDRs, and is also often responsible for most of the interactions with the peptide. The choice of a sequential approach, where loops are modeled individually was dictated by the need to sample reasonably sized conformational spaces [26].

### Modeling failures

In non-successful cases, either CDR loops were unusually long (11 residues for 1kj2 CDR3β) or the accessible conformational space of the loop was particularly large. Indeed, for the most notable failures, the *n/D_N-C_* values are 0.98, 1.28 and 1.22 Å^−1^ for 1mi5 CDR3α, 1kj2 CDR3β and 1nam CDR3β, respectively (see Table 1). As mentioned earlier, high values of *n/D_N-C_* are associated with a larger conformational space to sample.

In these cases, the small number of hydrogen bonds made with the pMHC in the crystal structure (0 or 1) also suggested that CDR loop conformation is determined by other types of interactions (mainly non polar) with the pMHC or interactions within the TCR itself [27] [28].

### NY-ESO-1 TCR repertoire structure prediction

Using our approach, we predicted six TCRpMHC structures of specific TCR recognizing NY-ESO-1/HLA-A*0201. Previous studies have already stated important characteristics of the interaction between TCR and pMHC in this repertoire. Experimental alanine scanning and *in silico* binding free energy calculations pointed out the importance of the central Met4/Trp5 residues of the NY-ESO-1 peptide in the recognition by TCR bearing the BV13 chain [7]. Also, Sami *et al.* [22] observed from recently published crystal structures of one given TCR (pdb 2PYE and 2PYF) that the CDR3β is subject to an important structural rearrangement upon complexation to NY-ESO-1/HLA-A2 and that CDR3α and β adopt a *lock* conformation around the peptide Met4 and Trp5. Our models showed this lock conformation for all the analyzed TCR of the repertoire. We further identified a conserved central Gly residue in the CDR3β sequence fitting into the notch formed by the Trp5 (Figure 5) and playing a key role in this *lock* conformation. Indeed, dramatic structural rearrangements were observed upon *in silico* mutations of the Gly to small residues Ala and Ser, which suggested that the proper *lock* conformation of the CDR3α and β was not accessible anymore. Experimental titration ELISA of soluble TCR and pMHC as well as measurements by flow cytometry of multimer binding at the surface of T-cells of the Ala and Ser mutants actually showed a total loss of binding affinity of the TCR to NY-ESO-1/HLA-A2 (Figure 6), which may be explained by the particular role played by the Gly residue in our model.

Possible explanations for the structural rearrangement and the loss of affinity upon complexation include: (i) the available space in the notch formed by Trp5 is not sufficient to accept any side chain, thus preventing the *lock* conformation of the CDR3 to occur. (ii) the Gly residue may provide improved backbone flexibility to the CDR3, as discussed by McCormack *et al.* [29] and Huang *et al.* [30], because of the wider range of accessible combinations of ϕ and ψ angles to Gly relative to other residues. This might be the key allowing the CDR3 to structurally rearrange upon complexation. Preliminary modeling results confirmed that the restriction of the Gly ϕ and ψ angles to values accessible to other amino acids prevented the CDR3 loop to adopt the lock conformation, and resulted in structural deviations that were comparable to the ones obtained by mutation to Ala and Ser (data not shown). The flexibility of the loop may also allow its residues to have access to a larger conformational space, in order to make optimal native contacts with the environment.

In conclusion, the study of NY-ESO-1 binders with our approach led to the rapid identification of key information that was not evident at the sequence level. The agreement between experiment and *in silico* results illustrates the efficiency of our method for TCR repertoire analysis in identifying key structural aspects linked to function, paving the road to structure-activity relationships studies and rational TCR design.

## Materials and Methods

Two modules were used for the TCRpMHC structure prediction from the amino acids sequence. First, the homology module was used to build the overall TCRpMHC model, as previously described by our group [9]. The *ab initio* prediction was used next to refine the CDR loops that determine the TCR specificity for a pMHC complex (Figure 1). Computations were spread on a computing grid and minimal user input was required.

### Manual setup

We defined a database of X-ray crystal structures available for homology computations and for quality evaluation of the method (Table S1). The peptide sequence to model was manually aligned with the peptides of the library. The order in which CDR loops were refined by the *ab initio* prediction was defined manually to offer flexibility, with the option of simulating several CDR loops simultaneously. All subsequent steps were automatically executed.

### Automated workflow

The IMGT® [31] (IMGT®, the international ImMunoGeneTics information system® http://www.imgt.org) database provides the positions of the two first complementary regions (CDR1 and CDR2) for each TCR alleles. The method performed a one-by-one alignment of the V chains of the target with the alleles provided by IMGT® to match the target sequence with its corresponding allele and determine the position of the CDR1 and CDR2 in the target. The templates were structurally aligned together and the target sequence was then aligned to the fixed structural alignment to determine the position of the CDR3. CDR loops have by definition limited sequence homology between different TCR, and have thus to be modeled *ab initio*. Therefore, canonical restraints on the ϕ and ψ dihedral angles of CDR loops (mean value and standard deviation) were added to limit the conformational space accessible to a CDR loop during the modeling steps. Canonical restraints consider key residues in the TCR sequence together with CDR length to categorize CDR in canonical groups associated with dihedral angles values [5]. We expanded the table available in the literature with more recent crystallography data from TCR and TCRpMHC [8] (Table S2).

#### Homology modeling

When required (NY-ESO-1 TCR repertoire analysis), the alignment defined above together with the canonical restraints of CDR loops dihedral angles was used for the homology modeling of the TCRpMHC complex. The computation of homology models as well as the clustering method were conducted as described by our group in [9]. The whole TCRpMHC complex, including TCRVα, TCRVβ, peptide, MHC class I and β2-microglobuline, is built during this step and is used then as an initial condition for CDR *ab initio* modeling.

#### *Ab initio* prediction (Figure 1)

The CDR loops were refined successively according to the user-defined order. The first CDR (or group of CDR) was modeled, using either a crystal structure (assessment of the algorithm) or a homology structure (NY-ESO-1 TCR repertoire analysis) as initial condition (Figure 1.1). In this step, all the residues of the complex not belonging to CDR loops remained fixed in space. Canonical restraints were used as described above (Figure 1.2). The program distributed the simulations on a computing grid until it collected 2000 conformers with a Modeller *pseudo*-energy function lower than 500 (Figure 1.3). For each conformer of this set, potential hydrogen bonds between the TCR and the pMHC were identified. Then, 2000 new complexes were computed from the same initial structure but with the potential hydrogen bonds computed as additional restraints (Figure 1.4). Our aim was to bias the population of conformers towards the region of conformational space demonstrating TCR-antigen (i.e. pMHC) interaction. This procedure was iterated 2 more times: on iteration i+1, the hydrogen bonds occurring in at least 2×i% of the 2000 conformers were added as restraints to further restrict the conformation space accessible to the system. (Figure 1.4). The RMSD matrix of the last set of conformers allowed clustering the candidates (Figure 1.6 to 1.8) [9]. The RMSD cutoff of the algorithm was automatically adjusted to ensure that the biggest cluster contained at least 20 conformers. To select the final conformer, the TCRep 3D scoring function was computed for each conformer within the best mean Modeller pseudo-energy cluster as the sum of the occurrence of each of its potential hydrogen bonds between the TCR and the pMHC in the 2000 conformers set (Figure 1.9). If no conformer in that cluster made potential hydrogen bonds with the pMHC, the one with the lowest *pseudo*-energy was selected. If another CDR (or group of CDR) had to be modeled, this conformer was used as initial condition. Therefore, to model one CDR, 8000 complete TCRpMHC were generated and the clustering based on a 400×400 (or bigger) RMSD matrix was computed, leading to a total computation time of about 7 CPU days on recent CPU architecture. The parameters shown above for the iterative modeling with canonical and hydrogen bonds restraints were set to the maximal values allowing to obtain the results in a reasonable amount of time, taking account of the available computing power. Making 3 iterations after the first set of conformers was shown to improve the ability of the *ab initio prediction* to model hydrogen bonds accurately (see discussion and Figure 8).

### Databases

TCR variable α and β chains CDR1 and CDR2 positions were determined from IMGT® (http://www.imgt.org) [31]. In this paper, crystallographic structures are named according to their Protein Data Bank accession numbers (Research Collaboratory for Structural Bioinformatics Protein Data Bank, http://www.rcsb.org/pdb/) [12]. Crystal structures of TCRpMHC were selected after the review of Rudolph *et al.* [8] : 1ao7 [32], 1bd2 [33], 1g6r [34], 1kj2 [35], 1lp9 [36], 1mi5 [27], 1nam [28], 1oga [37], 2bnr [21] and 2ckb [38] (Table S1). Redundant structures were ignored in our test set, but they were used nonetheless for canonical groups categorization. Crystal structures of TCR and fragments of TCR were also included in the templates list for homology modeling and canonical groups categorization: 1b88 [39], 1bec [40], 1i9e [41], 1h5b [42], 1kb5 [43], 1ktk [44], 1nfd [45] and 1934.4 (not in PDB) [46].

### Molecular modeling software

The automated approach to model the TCRpMHC complex was programmed in Perl (http:www.perl.org) and simulations were distributed on a computing grid. The following software was used for specialized tasks. Sequence and alignments, homology and CDR loop modeling were performed by Modeller 9v5 software [10] (http://salilab.org/modeller/modeller.html), loop modeling used the method of conjugated gradients combined with molecular dynamics and simulated annealing [10], [26]. Jali [47] performed single sequences alignments with structurally aligned block of crystal templates (http://bibiserv.techfak.uni-bielefeld.de/jali/). Potential hydrogen bonds were identified with the HBplus software version 3.15 [48].

### Protein production and Titration ELISA

The alpha and beta chains of TCR LAU 155 (AV23.1 & BV13.1), up to and including constant region residues alpha-Cys209 and beta-Cys242, were cloned into pHYK8 under the control of a CMV promoter. Similar to Chang et. al. [49], chain pairing was facilitated with an acidic-basic zipper, and a His-tag was included at the carboxy terminus of the beta chain. The mutation beta-Gly96Ser was introduced using the QuickChange® mutagenesis kit (Stratagene, La Jola, CA). Soluble TCR was produced at the Protein Expression Core Facility of the Ecole Polytechnique Fédérale de Lausanne in PEI transfected HEK 293 cells cultured over 5 days, and was subsequently purified with Ni-NTA agarose (Qiagen, Valencia, CA) and imidazole elution. Recombinant soluble HLA H and β2 microglobulin chains (HLA-A2) were obtained using a prokaryotic expression system (pET; R&D Systems, Minneapolis, MN) as previously described [50]. The chains were folded by dilution in the presence of NY-ESO-1_157–165_ peptide (p) and subsequently purified by fast protein liquid chromotography. The BirA enzymatic site, included at the carboxy terminus of the H chain, was then biotinylated (Avidity, Denver, CO). Protein quality was assessed by SDS-PAGE and concentrations were determined by Bradford measurement. Titration ELISA was used to assess TCR binding. Briefly, biotinylated pHLA-A2 was captured in streptavidin-coated plate wells blocked with 2% bovine serum albumin (BSA) in Tris buffered saline (TBS, pH 7.4). Soluble TCR, titrated in TBS, 1% BSA, 0.1% Tw, was incubated for 1.5 h at RT. TCR bound to the pHLA-A2 was detected with anti-b chain TCR MAb (Pierce, Rockford, IL; TCR1151) diluted 1/1500, followed by HRP-conjugated-goat- (anti-mouse IgG)-Ab (Pierce, 31430), diluted 1/1500 in TBS, 0.1% Tw, and HRP detection with ABTS in a citric acid and phosphate solution containing H_2_O_2_. Plate wells were thoroughly washed at each step. Plate readings were taken at OD_405–490 nm_.

### Lentiviral production, cell transduction and flow cytometry analysis

The Gly96Ala substitution was introduced into the wild-type (WT) TCR BV13.1 (patient LAU 155) DNA by PCR mutagenesis using the QuickChange mutagenesis kit (Strategene, La Jolla, CA) and confirmed by DNA sequencing (GenBank accession number: JN180298). Lentiviral vectors were produced by transient transfection of 293T cells, a human embryonic kidney (HEK) epithelial cell line that expresses the SV40 large T antigen (ATCC) [17], using a standard calcium phosphate precipitation protocol as described elsewhere [17]. In brief, 293T cells were cotransfected with the vector of interest (pRRL-hPGK-TCR Vα23.1-IRES-TCR Vβ13.1) and the transfer vector, envelope, and packaging plasmids (pRSV-Rev, pMD2-VSV-G, and pMDLg/pRRE). Supernatants were harvested 24 h and 48 h post transfection, filtered, and concentrated by ultracentrifugation. Pellets were resuspended in sterile cold PBS and directly used. A total of 1×10^6^/ml of SUP-T1 cells were transferred to pretreated polybrene plates (1 µg/ml) and transduced with concentrated lentiviral supernatant. After 5 days of culture in RPMI 1640 (Invitrogen) supplemented with 10% fetal calf serum, 10 mM Hepes, and antibiotics, cells were analyzed by flow cytometry using PE-labeled HLA-A2/NY–ESO-1_157–165_ (SLLMWITQA) multimer, FITC-conjugated antibody against human BV13.1 or CD8-PE-Cy7.

### Additional software

P-values were computed by the GraphPad Prism software. Molecular representations were made using the Visual Molecular Dynamics (VMD) software [51].

## Supporting Information

Figure S1 (A) Crystal structure of 1fo0 CDR3α (red transparent), displayed with its crystal neighbor TCRpMHC complex. The residues making the crystal contact, e.g. CDR3α Tyr97 (red) and MHC Ala135 (green) are shown in sticks. (B) TCRep 3D model of 1fo0 CDR3α (red transparent) demonstrates a significant conformational deviation from the crystal with a hydrogen bond between CDR3α Tyr97 (red) and Asp4 of the peptide (green).
(EPS)

Figure S2 Ramachandran plot for 2000 conformers of 1kj2 CDR2β Arg50, in unrestrained simulations, and with canonical restraints. The region defined by the canonical restraints is localized by an orange line. Ramachandran positions of the residues in the crystal structures are shown by an orange dot in each plot. Colored surfaces correspond to the ϕ/ψ accessible areas for all amino-acids except Gly. The plots are generated by the VMD software.
(EPS)

Figure S3 Success of CDR loop modeling is independent of conformational changes upon TCR binding to pMHC. RMSD of all predicted single-loops of the test set plotted against the corresponding RMSD between the bound and unbound crystal structures [25] (when available).
(EPS)

Table S1 (A) List of TCRpMHC crystal structures used in the test set. (B) List of crystal structures of unbound TCR and pMHC used to complement the template list and update the definition of canonical restraints.
(XLS)

Table S2 Table of the canonical groups defined by Al-Lazikani *et al.* [5]. Angles and SD values were updated with more recent structures (see Methods). Key residues are highlighted. The canonical group α2-1 is entirely defined by residues that are external to the CDR (e.g. Phe 32 and Leu 66).
(XLS)
